# Enhancing the X-ray Sensitivity of Cs_2_AgBiBr_6_ Double Perovskite Single Crystals through Cation Engineering

**DOI:** 10.1021/acsaom.4c00265

**Published:** 2024-09-26

**Authors:** Donato Valli, Heng Zhang, Marián Betušiak, Giacomo Romolini, Arne Meulemans, Daniel Escudero, Sudipta Seth, Qing Zhao, Zonglong Zhu, Mischa Bonn, Eduard Belas, Roman Grill, Hai Wang, Johan Hofkens, Elke Debroye

**Affiliations:** † Department of Chemistry, KU Leuven, Celestijnenlaan 200F, Heverlee 3001, Belgium; ‡ Max Planck Institute for Polymer Research, Mainz 55128, Germany; § Institute of Physics, Faculty of Mathematics and Physics, Charles University, Ke Karlovu 5, Prague 2 CZ-121 16, Czech Republic; ∥ School of Physics, Peking University, Yiheyuan Road No. 5, Haidian District, Beijing 100871 China; ⊥ Department of Chemistry, City University of Hong Kong, Kowloon, Hong Kong 999077, China

**Keywords:** perovskite, crystal, X-ray, doping, detector

## Abstract

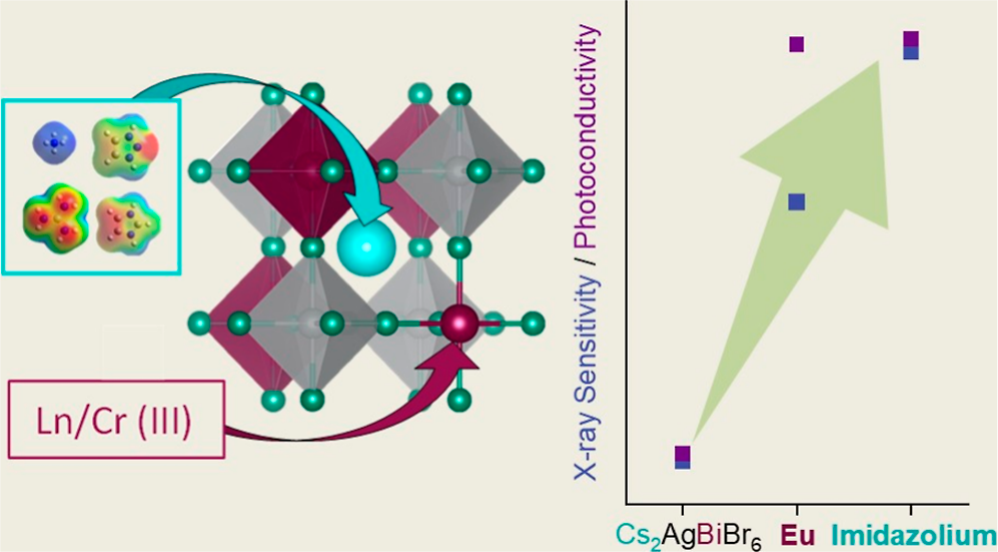

Owing to their outstanding optoelectronic properties, halide perovskite (HP) materials have been employed in a wide range of applications, including solar cells, light-emitting devices, and X-ray detectors. Among them, lead-free double HPs are characterized by enhanced stability and reduced toxicity compared with lead-based alternatives. Cs_2_AgBiBr_6_, in particular, has emerged as a promising candidate for direct X-ray detection. The detection sensitivity, on the other hand, cannot yet compete with that of lead-containing perovskites. Developing schemes to improve X-ray detection efficiency is critical for reducing radiation exposure in medical imaging applications. Here, we investigate the potential of controlled doping and cation substitution with either lanthanides or small organic cations to improve the X-ray detection performance of Cs_2_AgBiBr_6_. Our findings reveal that by growing the perovskite in a slightly Bi-poor and Eu-rich environment, the X-ray sensitivity significantly increases 7-fold (from 17 to 120 μC Gy_air_^–1^ cm^–2^) and simultaneously improves the phototo-dark current ratio (from 2.5 to 29). Additionally, Cs-site substitution with imidazolium remarkably enhances the sensitivity over 10-fold (180 μC Gy_air_^–1^ cm^–2^), and ammonium enhances the phototo-dark current ratio to 37. Terahertz photoconductivity measurements reveal a positive correlation between enhanced X-ray sensitivity and improved charge transport properties (e.g., increased scattering time and, thus, carrier mobility) by doping. This study outlines straightforward strategies for boosting X-ray detection and fundamental photoconductivity in lead-free double HP, with potential implications for broader optoelectronic applications.

## Introduction

Halide perovskite (HP) materials have witnessed a tremendous popularity in recent years due to their remarkable optoelectronic properties,^1−4^ with extensive applications ranging from solar cells to light-emitting devices, photocatalysts, and photodetectors.^5−10^ A cornerstone feature of HPs is their high degree of compositional tunability, which can be easily accessed by varying their atomic constituents.^4^ Pb-HPs feature a chemical structure of APbX_3_ where A is usually an (in)organic cation, and X is a halide anion. Replacing Pb^2+^ by a couple of heterovalent atoms (B, B′) can form double perovskites (A_2_BB′X_6_). Among these, the Cs_2_AgBiBr_6_ double perovskite is a promising candidate for X-ray detection owing to its robust chemical and optoelectronic properties. These properties include relatively high charge carrier mobility, large diffusion lengths, and low ionic migration, which jointly contribute to high dark resistivity and low dark-current coupled with its intrinsic high chemical stability.^3,11−14^ On top of that, its high average atomic number leads to a large X-ray absorption coefficient.^12,15,16^ It is worth emphasizing that a positive correlation between elevated radiation dosage and DNA damage has been established.^17,18^ As a result, the employment of a material with enhanced X-ray sensitivity, such as Cs_2_AgBiBr_6_, has the potential to deliver highly sensitive X-ray detectors requiring lower radiation doses due to its reduced leakage current (*I*_d_). This will not only improve device stability but also minimize the risk of DNA damage, offering safer options for medical imaging applications. Despite these advantages, the full potential of Cs_2_AgBiBr_6_ as a photoactive material in direct X-ray detection has not been fully exploited as a viable competitor against lead-based HP photodetectors.

**Figure 1:**
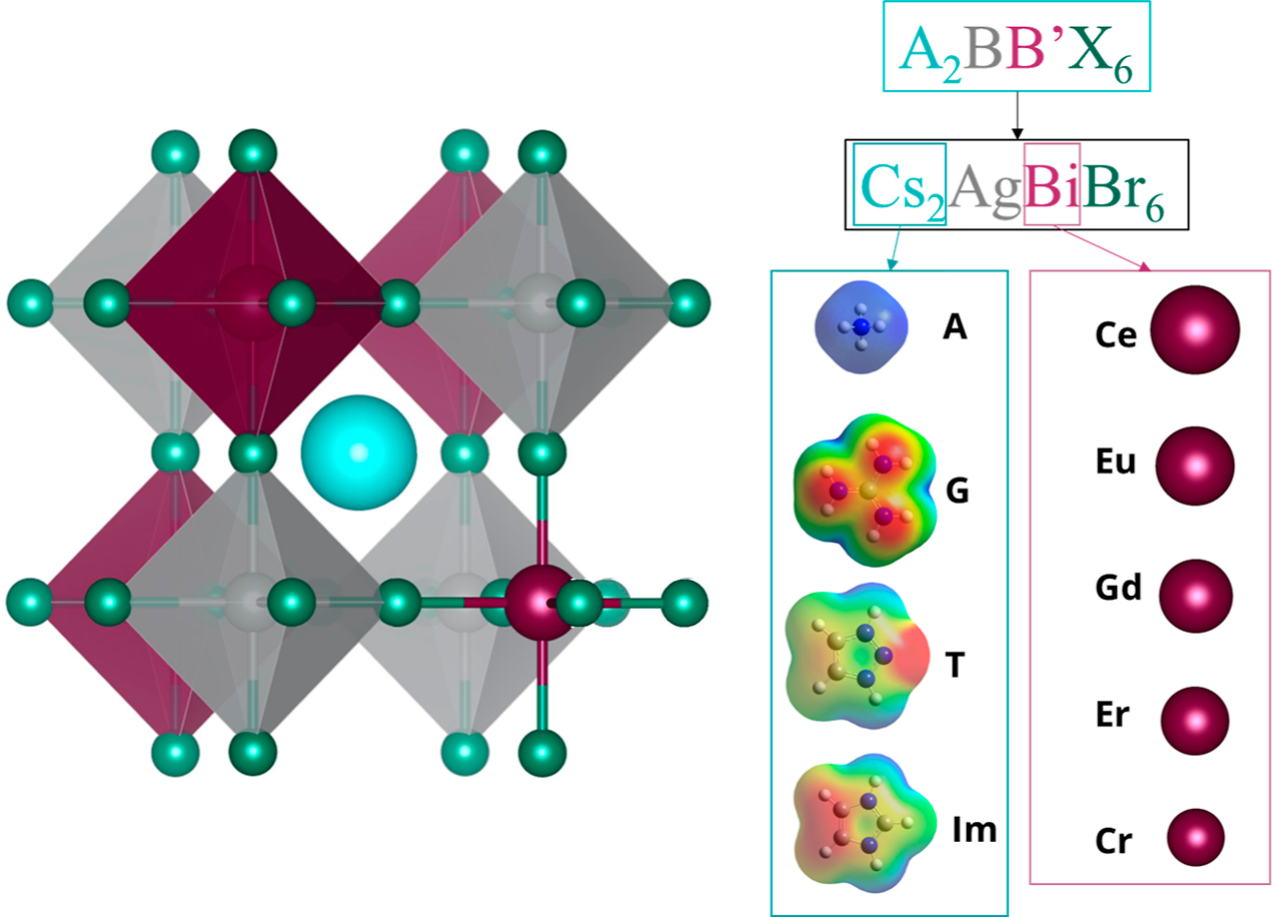
Schematic illustration of the Cs_2_AgBiBr_6_ crystal structure (left) and site-specific substitutions (Cs-site, cyan, and Bi-site magenta) (right). Cs-site substitutions are ammonium (A), guanidinium (G), triazolium (T), and imidazolium (Im).

One way to improve material properties, and specifically the X-ray sensitivity of perovskites, is to introduce atomic dopants in one of the A, B, or B′ crystallographic sites. By partially substituting Cs^+^ with Rb^+^ at the A-site, an increase in the material’s sensitivity to X-ray radiation by 4.5 times has been demonstrated by Keshavarz and Debroye et al.^12^ As for the B′-site, given its octahedral coordination, it offers the perfect chemical environment for lanthanide doping. It has already been proven to be effective in boosting the luminescent performance of other lead-free double perovskite nanocrystals, specifically Cs_2_AgInCl_6_, Cs_2_AgBiCl_6_, and Cs_2_AgBiBr_6_.^19−22^ In this work, we extend the study from Keshavarz and Debroye et al.^12^ by screening the impact of the controlled addition of lanthanides (III), Cr(III), and small monovalent organic cations, either at the bismuth Bi^3+^ or Cs^+^ crystallographic sites,^23,24^ specifically, Ce^3+^, Eu^3+^, Gd^3+^, Er^3+^, and Cr^3+^ and ammonium (A), guanidinium (G), triazolium (T), and imidazolium (I) (Figure 1). To understand the impact of atomic substitution on electrical properties, we comprehensively characterized the key electrical transport or device operation parameters, including: X-ray detection sensitivity (*S*), photocurrent (*I*_ph_), and dark-current (*I*_d_), as well as their ratio (*I*_ph_/*I*_d_), photoconductivity (PP), charge carrier scattering time (τ_s_), photocurrent density (*J*_ph_), and dark current density (*J*_d_). The results highlight that introducing small amounts of dopants at either the Bi- or Cs-site can substantially enhance the X-ray detection performance of the Cs_2_AgBiBr_6_ material. This enhancement can be traced, at least in part, to improvements in the electrical mobility of charge carriers, as is evident from Terahertz (THz) spectroscopy. These insights are of fundamental physical relevance and directly linked to practical applications of double perovskite materials.

## Results and Discussion

**Figure 2:**
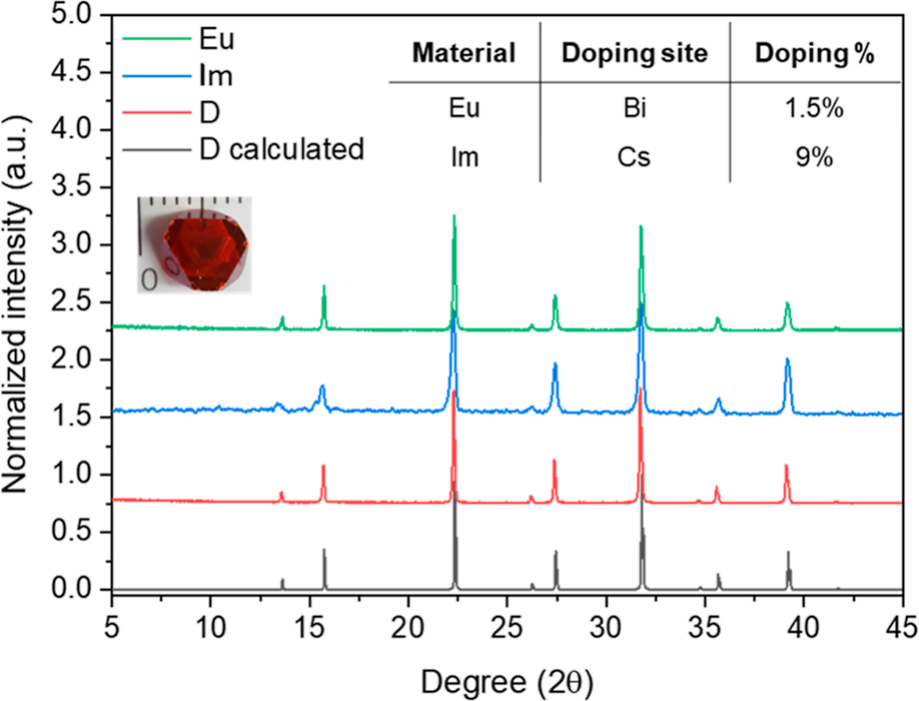
Calculated PXRD pattern for the Cs_2_AgBiBr_6_ structure (black), together with the PXRD patterns of the ground SCs (inset, mm scale) for the pristine material (red) and the best-performing doped materials at the Cs- (blue) and Bi-sites (green) by Im and Eu^3+^, respectively. The table provides the doping amounts corresponding to each dopant’s pattern.

We first present and discuss the impact of doping and ion substitution on the structural and optical characteristics of Cs_2_AgBiBr_6_ double perovskite, henceforth referred to as **D**. We simplify our references to the various doped materials by mentioning the foreign elements, such as using **Eu** for Eu doping and so on. Millimeter-sized single crystals (SCs) were synthesized following a supersaturated solution cooling method as previously reported,^15^ by modifying the starting solution composition. To assess the integration of substituents within the pristine material, inductively coupled plasma optical emission spectroscopy measurements were conducted, revealing 1–2% doping for Bi-replacement and between 9 and 14% substitution for Cs-substitutions, respectively (Figure 2). To assess the influence of doping on the crystal structures, powder X-ray diffraction (PXRD) patterns for both the doped and pristine materials were recorded. Variations in X-ray response can be attributed to changes in crystal structure or side-phase formation; notably, the trigonal Cs_3_Bi_2_Br_9_ phase may form alongside the Cs_2_AgBiBr_6_ cubic  phase.^25,26^ The PXRD patterns of double perovskites with various dopants are shown in Figure 2. The analysis employing LeBail fit confirmed that the pristine sample structure matches the cubic  phase. Furthermore, a low amount of dopants does not significantly affect the cubic symmetry of the crystal lattice or promote any side-phase formation, as evidenced by the absence of extra peaks upon doping. Negligible changes in the lattice parameters were found for Bi^3+^ replacement (Table S1), aligning with other reports.^12,19,20,27^ In Keshavarz et al., 2% Cs-to-alkali metal substitution leads to only a few thousandth of an Å shift in lattice parameter as assessed by Rietveld refinement on high-resolution XRD synchrotron data.^12^ The negligible shift can also be justified by noting that a full substitution of Bi^3+^ to Eu^3+^ while retaining the same cubic space group , i.e., shifting from Cs_2_AgBiCl_6_ to Cs_2_AgEuCl_6_, results in a difference of 0.05–0.15 Å (depending on the source).^28−33^ This finding highlights that more advanced analysis of minute amounts of any site-substitution in A_2_BB′X_6_ double perovskites could be useful. For Cs substitution with ammonium salts, although the cubic lattice symmetry remained unchanged, slight variations in the lattice parameters have been observed, which reflect the dimensions of the substituents. Smaller substituents, such as ammonium (ionic radius of 1.46 Å), result in a greater lattice contraction (11.14 Å) compared to Cs^+^ (ionic radius of 1.81 Å, lattice size 11.26 Å). In contrast, larger and nonspherical ammonium salts like guanidinium (2.78 Å) and imidazolium (2.58 Å) exhibit an increase in the lattice constant compared to ammonium, but they are still smaller than **D**. This behavior can be attributed to the differences in structural symmetry between the spherical Cs^+^ cation and the various ammonium ones (Figure S1). These structural differences might lead to a distinct reorientation of the flatter guanidinium, triazolium, and imidazolium cations within the cuboctahedral site when compared to the more symmetrical ammonium cation, which exhibits a tetrahedral symmetry. Additionally, the diverse hydrogen bonding patterns, derived from their dissimilar structures and atomic compositions of the organic cations, may also influence this behavior. Importantly, the absence of peaks at lower 2θ degrees rules out the possibility of 2D-layered substructure formation, with the organic cations acting as spacers between the Ag–Bi–Br octahedra inorganic sublattice upon Cs^+^ substitution.^34^ This observation confirms that the dopant occupies the cuboctahedral cavity of the Cs^+^ ion.

In addition to structural properties, we explored the steady-state optical properties, of the various single-crystal materials, focusing on their absorption and emission characteristics (Figures S2 and S3). According to Klein’s rule, the ionization energy *W*_±_ is nearly 3 times the bandgap energy *E*_g_; a smaller bandgap could, therefore, increase charge generation yield per quantum of X-ray, following: Δ*Q* = *eE*_X-ray_/*W*_±_ (where Δ*Q* represents the amount of photogenerated charge, *e* is the elementary charge, and *E*_X-ray_ is the energy of the incident X-ray photon).^15^ The introduction of dopants led to a general slight decrease in the material’s bandgap for most samples (see the summary in the Supporting Information), which, with all other parameters being equal, may favor more efficient X-ray-to-electron conversion.

**Figure 3:**
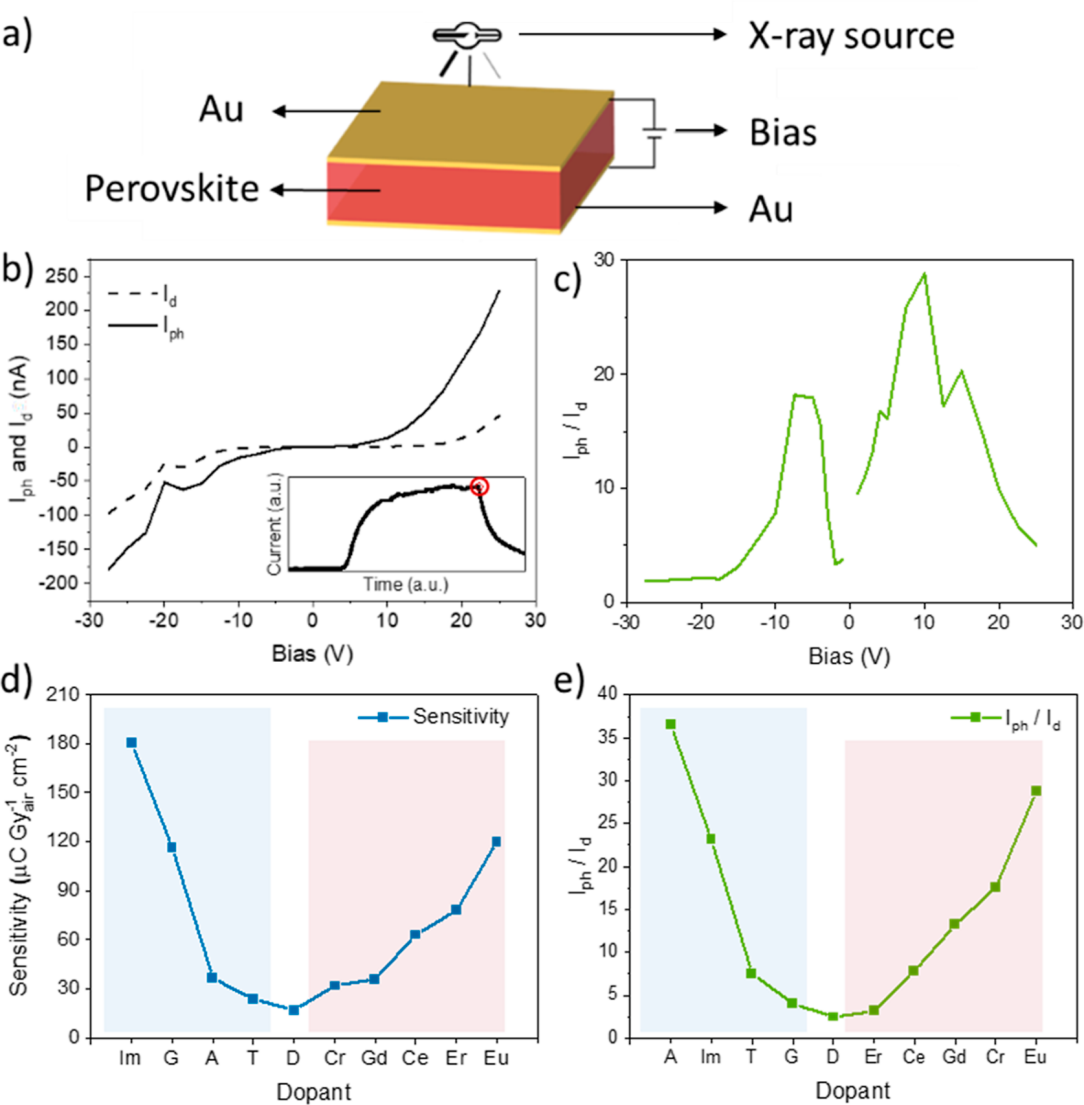
(a) Schematic representation of the device and X-ray photodetection setup. (b) Representative example of the *I*_ph_ and *I*_d_ curves for **Eu**, and current over time evolution (inset) where the red circle corresponds to the photocurrent value used for the following graphs in (c–e). (c) Resulting *I*_ph_/*I*_d_ ratio for sample **Eu** showing a peak at 10 V, which is the same bias used to calculate the sensitivities (d,e). Sensitivity and current ratio *I*_ph_/*I*_d_ ratios for the pristine (middle, labeled “D”) and doped materials (Cs-site cyan and Bi-site magenta shade).

After the structural and optical properties of the different materials were assessed, their X-ray responses were evaluated (Figure 3). To collect the voltage-dependent photo- and dark currents, *I*_ph_ and *I*_d_ (Figure 3b), and later extract important photoelectrical parameters (Figure 3c–e), the top and bottom surfaces of the SCs were coated with a gold layer. Prior to the analysis of the X-ray related parameters, the transient photocurrent behavior of the material has been assessed in order to get a deeper understanding of its fundamental photoelectrical characteristics. As shown in the inset of Figure 3b, the test devices show saturation behavior of the X-ray-generated photocurrent deviating from the ideal flat current response. Usually, such a photocurrent line shape can be split in two characteristic processes: (i) initial transient peak with follow-up damping or (ii) slow rise of the photocurrent to the saturation. A comprehensive review of the phenomena and a generic model may be found in the following report from Bisquert et al.^35^ Here, we propose an original model considering the charging of mobile ions contributing to the electric current, which captures the exponential-like evolution of the recorded photocurrent in highly resistive samples. Details of the model and simulations (Figure S4) are presented in the Supporting Information. A key point of this model is the assumption of a mobile defect—a deep donor hole trap, probably a native defect—positioned below the middle of the band gap (estimated to be about 0.65 eV above the valence band). In the highly resistive samples considered in this study, the Fermi energy is localized above the donor. This will result in the donor being neutral; hence, it does not participate in charge transport. In contrast, in low resistive samples (characterized by a step-like increase in photocurrent), the Fermi energy is below the donor, rendering the donor positively charged and capable of drifting under an electric bias.^36^ Upon X-ray excitation, the quasi-Fermi energy shifts toward the valence band, causing the donor level to charge even in highly resistive samples. The step-like increase in photocurrent in low-resistive samples is induced by free carriers excited by X-rays, where an ion drift contributes to lowered resistivity regardless of the excitation. Conversely, the gradual increase of photocurrent in highly resistive samples is induced by the continuous charging of donor level due to trapping of photoexcited holes and its contribution to *I*_ph_. This is in line with what would be expected for the pristine double perovskite and is also demonstrated for the doped materials in this study. For precision, the slow evolution of the photocurrent over a range of seconds can be simulated with a hole-trapping cross section of 3 × 10^–16^ cm^2^, being observed in semiconductors within the usual range.^37^ The model also consistently explains the specific features of the proportionality of *I*_ph_ and *I*_d_ observed in most samples. While in the highly resistive samples, the donor level is filled by electrons and can trap photoexcited holes to reduce the hole lifetime, donors in low resistive samples are positively charged and do not trap holes whose lifetime remains large.

Sensitivity (eq 1, where A is the illuminated area and D the dose rate) is a crucial parameter in X-ray detection, as it describes the device’s ability to detect and convert ionizing radiation into an electrical signal.
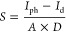
1

Figure 3d presents the trend of *S* upon Bi-site replacement. Notably, this analysis shows that Eu-doping, among the different lanthanides tested, is a very promising dopant for enhancing the X-ray sensitivity (120 μC Gy_air_^–1^ cm^–2^) up to a 7-fold increase compared to the pristine **D** SC (17 μC Gy_air_^–1^ cm^–2^). However, there is no clear linear trend when increasing the atomic number in the lanthanide series. The initial monotonic increase that follows the lanthanide series—**Ce** and **Eu**—is interrupted by the lower *S* of **Gd** and a subsequent increase for **Er**. Anyway, the overall increase in *S* can be partially attributed to the fact that lanthanide cations possess a K-edge energy value of around 50 keV (which corresponds to the binding energy of the K-shell electrons), which is smaller than that of Bi (90 keV; Table S2). This might lead to scintillation followed by reabsorption and conversion to photocharges in the doped perovskites compared to the pristine **D** sample. Besides *S*, *I*_ph_/*I*_d_ is another critical parameter when evaluating X-ray detectors, as it reports the device’s ability to distinguish between incident X-ray photons and background noise (Figure 3e). A high *I*_ph_/*I*_d_ is desirable and allows for the operation of X-ray imaging on the low-dose end. **Eu** demonstrated a peak ratio among the different lanthanides with an exceptional *I*_ph_/*I*_d_ value of 29 with an 11.6-fold improvement over pristine **D** (2.5), further underlining its promise as a dopant for enhancing the overall detector performance. Besides the lanthanide series, transition-metal **Cr** also shows an increase in *S* (31.8 μC Gy_air_^–1^ cm^–2^) and *I*_ph_/*I*_d_ (18). This finding highlights the potential of transition-metal dopants, such as Cr^3+^, in affecting the optoelectronic characteristics of the **D** material, particularly for photodetection and photovoltaic applications, while other transition metals like Cu, Fe, and Ru have a more important role in IR light detection.^38−40^ Considering the *J*_d_ and *J*_ph_ values at 10 V (Figure S5), the same trend as for the *S* values is found, i.e., **Eu** > **Er** > **Ce** > **Gd** > **Cr**. However, due to a different contribution to *J*_d_ and *J*_ph_ from every single dopant, it turns out that the *I*_ph_/*I*_d_ ratio eventually slightly deviates from the *S* trend. Notably, while **Eu** achieves a maximum *I*_ph_/*I*_d_ value, **Er** doping significantly increases the overall conductivity by enhancing *J*_ph,d_ in comparison to the pristine sample **D** (Figure S5).

**Figure 4:**
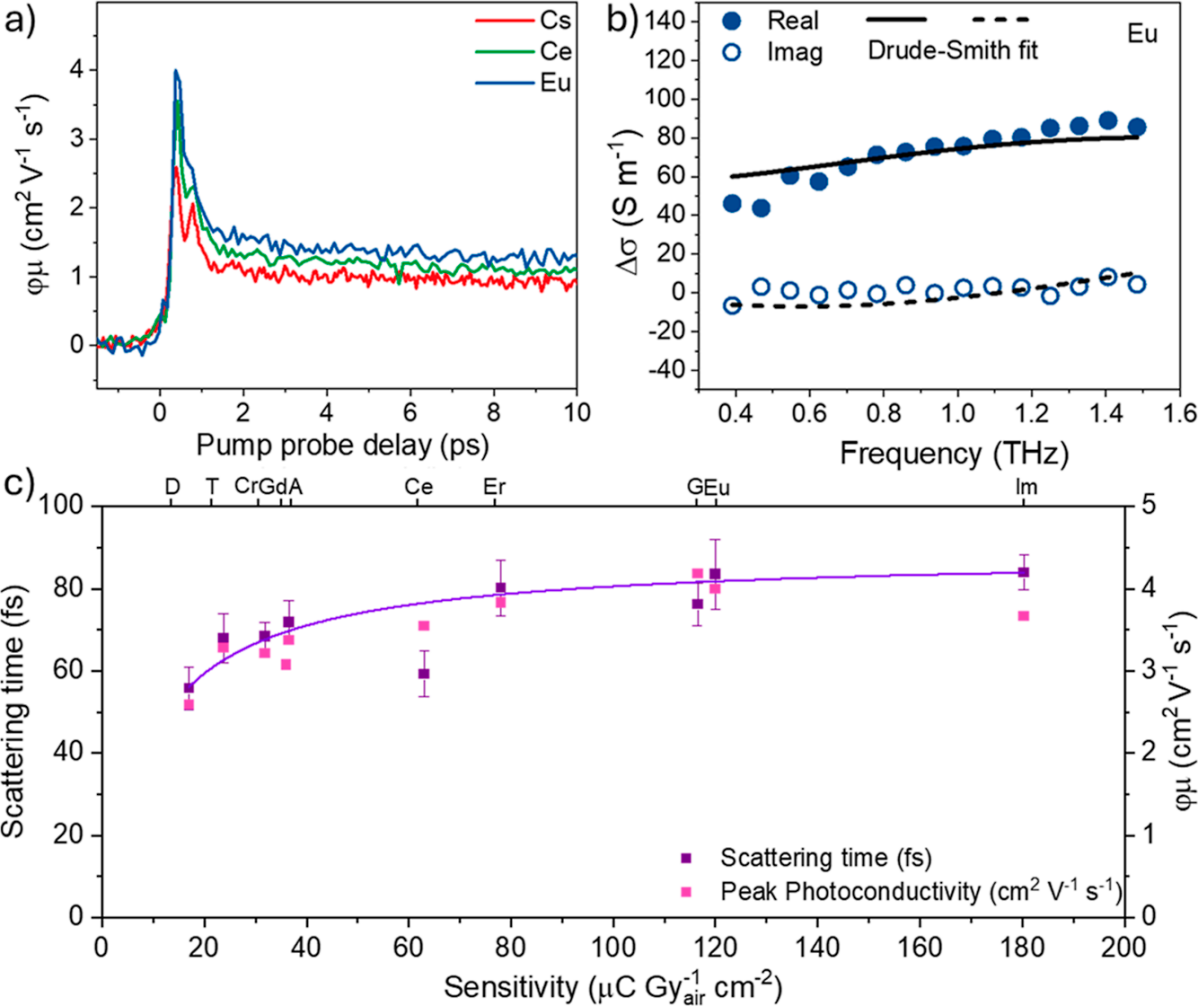
(a) Photoconductivity dynamics normalized to the absorbed photon density for **Eu**, **Ce**, and **Cs** measured with pump fluence of 25 μJ/cm^2^ at 400 nm. (b) Photoconductivity spectrum of **Eu** recorded at a pump–probe delay time of 40 ps. (c) Correlation plot among the X-ray sensitivity values (*x*-axis) by device measurements, and scattering time (left *y*-axis) and the peak photoconductivity (right *y*-axis) by THz spectroscopy. The solid line is a guide to the eyes.

Beyond conventional photoelectrical characterizations, we performed optical pump-terahertz probe (OPTP) measurements to correlate the macroscopic device performance with the microscopic charge transport properties (Figure 4). OPTP provides a noninvasive all-optical approach to interrogate ultrafast charge carrier dynamics and charge transport effects. To this end, an optical pump pulse (400 nm, 25 μJ/cm^2^) was employed to photoinject electrons in the conduction and holes in the valence band of the double perovskites.^12^ A single-cycle THz pulse, as an ultrafast oscillating electrical bias, is then employed as the probe to interact with photogenerated charges. Free carriers absorb the THz field, leading to its attenuation. The relative attenuation of the THz electric field −Δ*E*/*E* is proportional to the photoconductivity Δσ, where *E* is the THz field through the unpumped sample. Figure 4a presents the photoconductivity dynamics (per absorbed photon density, *N*_abs_) of bare **D** and Eu- and Ce-doped samples. In line with X-ray detection experiments, Eu- and Ce-doped samples deliver higher photoconductivity.

By Fourier transformation of the time-domain THz field, one can further obtain the frequency-resolved photoconductivity spectrum, as exemplified in Figure 4b for Eu-doped double perovskite at a pump–probe delay time of 40 ps. The photoconductivity spectrum can be well fitted by the modified Drude model, i.e., the Drude–Smith model (2), which reads
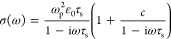
2Here, ε_0_ is the vacuum permittivity, ω_p_ is the plasma frequency, ω is the angular frequency, and τ_s_ is the momentum scattering time. The so-called “*c* parameter” (−1 ≤ *c* ≤ 0) was introduced into the original Drude model to account for the backscattering of the charge carriers from, e.g., grain boundaries or interfaces.^41^ In Figure 4c, we correlate the extracted scattering time τ_s_ and photoconductivity peak with the X-ray sensitivity for all investigated samples. First of all, the extracted scattering times follow the same trend of the peak photoconductivity amplitude (per *N*_abs_). As Δσ = *N*_abs_ φ*e*μ (with φ as the free carrier generation quantum yield), this result suggests that the change in the momentum scattering time or further carrier mobility, other than effective mass, dictates the photoconductivity trend. More importantly, our THz results show a positive correlation with the X-ray sensitivity *S* of all of the investigated samples. This result strongly suggests that the enhancement in *S* primarily arises from the increased charge mobility, specifically the improvement in scattering time denoted as μ = *e*τ_s_/m*. This intriguing observation may be rationalized by a scenario in which introducing a proper amount of dopants into a double perovskite lattice could passivate defects, thus reducing the charge scattering events. The scattering of charge appears to be limiting at low scattering times (τ_s_ ≲ 70 fs), while for larger scattering times, the τ_s_–*S* curve in Figure 4c levels off, and the sensitivity appears to be limited by factors different from the charge mobility.

As quaternary compounds, double perovskite materials inherently possess many competing binary and ternary side phases as well as a relatively large number of potential point defects that can form. However, the positive decomposition enthalpy for **D** makes side-phase formation rather difficult, as also demonstrated by the previously reported structural analysis. Regarding defects, halide vacancies require relatively low energy to form, while interstitials involving other elements in the structure require considerably more energy due to the closely packed structure, especially for ionic charges higher than +1 (e.g., the Bi-site dopants). Antisite defects due to the same octahedral coordination of Ag and Bi can usually be formed. It has also been reported that Bi-poor, Br-rich conditions, and low growth temperatures below 210 °C are effective in suppressing bromide vacancies (V_Br_).^42,43^ It is important to emphasize that our synthetic protocol meticulously adhered to these conditions: partial substitution of Bi^3+^, utilization of concentrated HBr as the synthetic medium, and strict temperature control to maintain synthesis below 120 °C. The partial substitution of Bi^3+^ can also avoid the orbital hybridization between Ag^+^ and Bi^3+^ in the case of V_Br_. Moreover, the vast photochemistry of the lanthanides elements, deriving from their f–f and f–d transition, which has proven to be effective in up-conversion phosphors, might also play a role in photon recycling and detrapping processes in the perovskite lattice hosting them.^44^ For instance, the potential alignment of lanthanides’ orbital energy levels with the shallow defect states could contribute to mitigating detrimental defect-induced recombination.^45^ All these effects could play a significant role in terms of the device performance, for instance, the nearly 1 order of magnitude improvement in the sensitivity of the X-ray detection for the imidazolium-doped sample.

Shifting to Cs substitution, it can be seen that there is an increase in *S* from triazolium to ammonium, guanidinium, and imidazolium. Among the ammonium salts, imidazolium is found to yield the highest sensitivity value, reaching 180 μC Gy_air_^–1^ cm^–2^, while the highest *I*_ph_/*I*_d_ ratios are observed for ammonium (37), followed by imidazolium (23). Conversely, the other two dopants, guanidinium and triazolium, do not effectively enhance *I*_ph_/*I*_d_, primarily due to their relatively higher *J*_d_ values (Figure S5). Notably, the lowest *J*_d_ value for ammonium strongly contributes to the resulting high *I*_ph_/*I*_d_ value, while the slightly higher *J*_d_ value for imidazolium, leads to a lower ratio, even though imidazolium has the highest *J*_ph_ among the different ammonium salts. In this particular instance, it becomes evident that *J*_d_ suppression plays a crucial role in influencing the performance of *I*_ph_/*I*_d_, as opposed to a significant increase in the level of *J*_ph_ in relation to that of *J*_d_. Furthermore, it is noteworthy to observe that when comparing Cs-substitution with ammonium cations to Rb-doping (4.5 times increase in *S* as compared to the pristine composition), an even higher enhancement in X-ray-related parameters is also evident.^12^ In this case, the peak value for imidazolium is nearly 1 order of magnitude higher than that of **D** and almost double in comparison to Rb doping. However, similar to the effects of Rb-doping, the combination of reduced *J*_d_ and increased *J*_ph_ contributes to the heightened *S* values. Moreover, although Rb-doping does not yield substantial differences in terms of PP and τ_s_, these values resulted in being higher for all the observed ammonium cations. One possible explanation for these variations in behavior can be attributed to the inherent chemical distinctions between the inorganic cation, Rb^+^, and the organic substituents. The latter not only lack the spherical symmetry possessed by the rubidium cation but also can form hydrogen bonds within the structure.

Notably, triazolium, guanidinium, and imidazolium possess aromatic systems, and triazolium and imidazolium exhibit a dipole moment. Despite the relatively modest impact of ammonium on *S*, it demonstrates significant benefits in terms of the *I*_ph_/*I*_d_ ratio. This can be attributed to its smaller size, which induces the highest lattice contraction, as confirmed by the PXRD measurements. This contraction likely promotes better orbital overlap between Ag and Bi ions and the surrounding Br ions.^46^ Additionally, ammonium can undergo hydrogen bonding with bromide anions and thereby reduce ion migration, a key factor contributing to the dark current in HPs. For aromatic ammonium cations, although certain considerations related to hydrogen bonding remain pertinent, it is crucial to account for their inherent electron richness and anisotropic electronic distribution (as depicted in Figure S1). These factors can significantly impact the material’s dielectric constant, surpassing the influence observed with the A cation, thereby affecting the motion of charge carriers in the material. Furthermore, cyclic ammonium cations such as triazolium and imidazolium exhibit a permanent dipole moment, known to influence the properties of charge carriers by aiding in charge screening and facilitating efficient separation from bound excitons. In this regard, a parallelism can be drawn between the full-inorganic HP CsPbX_3_ and (CH_3_NH_3_)PbX_3_. Despite both possessing a high dielectric constant, the latter shows a considerable contribution to the dielectric constant from the methylammonium (MA) dipole rotation. As a consequence, at low frequencies, the dielectric constant of CsPbX_3_ amounts to almost half of that for MAPbX_3_, resulting in less efficient screening of charged defects and leading to reduced effective charge carrier mobility.^47^ So, this characteristic may contribute to lowering the recombination rate, ultimately allowing increased extraction of photogenerated charges in the A-site doped materials.^48,49^ Moreover, imidazolium and triazolium might be capable of interacting with both Bi^3+^ and Ag^+^ ions through their aromatic ring’s pi-system.^50^ When we consider all these factors together, we can argue that while ammonium primarily affects the structure of the **D** material and may suppress ion migration, the other cations also effectively stabilize the *D* structure. Moreover, the increase in τ_s_ is consistent with the plausible decrease in defect concentration, probably suppressing ion migration.

## Conclusions

In conclusion, our study offers valuable insights into the influence of various dopants and substituents on the X-ray detection performance of the Cs_2_AgBiBr_6_ double perovskite, emphasizing the potential of such “chemical engineering” to enhance X-ray-related and general optoelectronic properties. Lanthanide dopants at the Bi-site exhibited diverse effects on *S*, with **Eu** demonstrating the highest enhancement in X-ray detection performance. Notably, Cr-doping, despite not being a lanthanide, also displayed a significant increase in *S*. These findings were correlated to OPTP measurements, showing an increase in the photoconductivity and scattering time. It must be noted that the reported improved carrier dynamics are not necessarily the only responsible parameter for the marked increase in *S*. This work also points out several improvements in the enhancement of *J*_ph_, reduction in *J*_d_ (linked to the defect landscape and lower ionic migration), and phototo-dark-current ratio (*I*_ph_/*I*_d_). Similar considerations apply to Cs substitution with ammonium salts, where *S* was generally increased, with imidazolium exhibiting the highest value among all of the substitutions. The very high *I*_ph_/*I*_d_ ratio of the ammonium substituted sample demonstrated how lowering *I*_d_ is of foremost importance, paving the way toward lower detection limit X-ray sensors, which will eventually operate at lower X-ray doses. Further investigations could focus on a combined investigation of photoluminescence (PL) properties such as PL quantum yield and lifetimes in the ns to μs regimes and potentially spatial PL mapping, to further elucidate the underlying photophysical processes governing the increase in performance.^51^ Moreover, screening doping concentrations and exploring Cs–Bi codoping strategies could enhance the overall material performance potentially further. This would be even more effective if combined with a study on the impact of dopants in other synthetic processes governed by kinetic or thermodynamic control.^52^ It is important to note that these findings may have broader applications in optoelectronics, including fields such as solar cells.

## Experimental Section

### Material Synthesis

Cs_2_AgBiBr_6_ SCs were grown similarly to a previously reported procedure under ambient atmospheric conditions.^15^ Briefly, 1.0 mmol of BiBr_3_ (≥98%, Sigma-Aldrich), 2.0 mmol of CsBr (99.9%, Sigma-Aldrich), and 1.0 mmol of AgBr (≥99%, Chem-Lab) were added in 10.5 mL of HBr (reagent grade, 48 wt %, Acros Organics) resulting in a mixture. The precursor solution was modified to substitute 5% molar ratio to Bi for lanthanides and chromium bromides (CeBr_3_, 99.9%, Strem chemicals INC; EuBr_3_ hydrate, ≥99.99%, MERCK Life Science BV; ErBr_3_ hydrate, Bio Connect BV; GdBr_3_, 99.9%, Thermo Fisher GMBH) and 20% in molar ratio to Cs for the ammonium cation doping (ammonium bromide, 99+%, Acros organics; imidazolium bromide, 98%, Ossila LTD; triazole, guanidinium bromide, ≥98%, Sigma-Aldrich). After 10 min of sonication, the mixture with an orange precipitate on the bottom was heated to 120 °C for 3 h in order to obtain a clear supersaturated solution. Then, the solution was slowly cooled in the following controlled manner: (i) 2 °C cooling per hour to 100 °C, (ii) 1 °C cooling per hour to 50 °C, (iii) stable heating at 50 °C for 10 h, and (iv) 1 °C cooling per hour to 40 °C. During the last step SCs of the right dimensions 1 × 1 to 5 × 5 mm^2^ as the top surface were collected and then washed with isopropanol (HPLC grade, Sigma-Aldrich) followed by vacuum drying at 60 °C.

### Powder X-ray Diffraction

PXRD patterns were recorded with a Malvern PANalytical Empyrean diffractometer in transmission mode by using a PIXcel3D solid-state detector and a Cu anode. The diffractogram was recorded in the range of 2θ between 5 and 45°. The resulting X-ray diffraction patterns were refined through the FULLPROF program using the LeBail fit.^53^ The starting parameters for lattice and space group were taken from ref (54).

### UV–Vis Diffuse Reflectance Spectroscopy

Diffuse-reflectance spectroscopy data were recorded on a Lambda 950 UV–vis spectrophotometer (PerkinElmer) using BaSO_4_ powder as a white reference. The resulting diffused reflectance (*R*) was converted to *F*(*R*) = (1 – *R*)^2^/2*R* by using the Kubelka–Munk function. The indirect bandgap values were extracted by means of Tauc plot analysis.

### Steady-State PL

Emission spectra were recorded on an Edinburgh FLS980 fluorometer in front-face mode.

### Device Fabrication

SCs with flat surfaces between 1 × 1 and 5 × 5 mm^2^ were deposited, on parallel sides, 80 nm thick Au layers as the ohmic contact (Au/Perovskite/Au) using a thermal evaporator.

### X-ray Photodetection

The devices were exposed to X-ray photons from a tungsten anode X-ray tube (maximum X-ray photon energy 70 keV, peak intensity 30 keV) at 1 m, constant, distance from the source. A precise control over the X-ray dose (10.8 mGy s^–1^) was achieved by modulating the current flow in the tube and measured with a RaySafe Model Solo R/F dosimeter. A Keithley 2000 was used to control the bias voltage and record the resulting current with and without X-ray photons. Due to the broad range photodetecting capabilities of the perovskite materials, the X-ray photoresponses were collected in the dark.

### Photoelectrical Characterization Model and Simulations

Illustrative results of simulations based on the Shockley–Rad–Hall theory,^55,56^ neglecting spatial charge and free carrier distribution^57^ are presented in Figure S3. They clearly demonstrate both cases of slow and fast conductivity evolution. The simulations were performed at room temperature with the following set of model parameters: deep donor density *N*_D_ = 10^13^ cm^–3^, position of the level above valence band *E*_D_ = 0.65 eV, capture cross section of holes σ_h_ = 3 × 10^–16^ cm^2^, effective mass of holes *m*_h_ = 0.14*m*_0_, hole mobility μ_h_ = 25 cm^2^/(V s), and diffusion coefficient of charged donor *D* = 6 × 10^–6^ cm^2^/s. The hole density in the dark *p*_d_ was chosen as 2 × 10^7^ and 2 × 10^8^ cm^–3^ in high-resistive and low-resistive samples, respectively. Hole densities correspond to the position Fermi energy 0.64 and 0.58 eV above the valence band, respectively. Hole densities after excitation *p*_ex_ were 4 × 10^7^ and 2.8 × 10^8^ cm^–3^ in high resistive and low resistive samples, respectively. Although most of the parameters were guessed casually, they agree with similar data found in the literature.

### Electrostatic Surface Potentials

Electrostatic surface potentials (ESP) for the different ammonium cations (Figure S1) were determined with density functional theory (DFT) calculations using the B3LYP functional and 6-311+G(d,p) basis set.

### Confocal Microscopy

Confocal microscopy images of the pristine and Cs- and Bi-site-doped representative materials (Im and Eu respectively) were recorded on a Leica TCS SP8 X confocal microscope. The SC’s signals were recorded through a 10× air objective, under a laser excitation of 405 nm and 65 μW laser power.

## Supporting Information Available

Additional information regarding experimental procedures and results; lattice parameters of pristine and doped samples; calculated ESP for the ammonium cations and their partial atomic charges and dipole moments and explanation about the effect of the obtained bandgap shift on the X-ray sensitivity; normalized Tauc plots and emission spectra together with the calculated bandgaps of the pristine and doped samples, stereomicroscopy and confocal microscopy images of pristine and doped SCs; explanation for the model of photoconductivity evolution for high and low resistive samples; simulation results and Fermi levels; K-edge energies of the lanthanides dopants; and photo- and dark-current densities of the pristine and doped samples (PDF)
