# Supplementary material for: Enhancing the X-ray Sensitivity of Cs2AgBiBr6 Double Perovskite Single Crystals through Cation Engineering
Source: ACS Appl Opt Mater. 2024 Sep 26;2(10):2075–84. doi: 10.1021/acsaom.4c00265 (PMC11519909; doi:10.1021/acsaom.4c00265)
Supplement: Supplementary file 1 — ot4c00265_si_001.pdf [file ot4c00265_si_001.pdf]

# Supporting Information

## Enhancing the X-ray sensitivity of $Cs_2AgBiBr_6$ double perovskite single crystals through cation engineering

Donato Valli[a], Heng Zhang[b], Marián Betušiak[c], Giacomo Romolini[a], Arne Meulemans[a], Daniel Escudero[a], Sudipta Seth[a], Qing Zhao[d], Zonglong Zhu[e], Mischa Bonn[b], Eduard Belas[c], Roman Grill[c], Hai Wang[b], Johan Hofkens[a], Elke Debroye*[a]

[a] Department of Chemistry, KU Leuven, Celestijnenlaan 200F, 3001, Heverlee, Belgium

[b] Max Planck Institute for Polymer Research, 55128 Mainz, Germany

[c] Charles University, Faculty of Mathematics and Physics, Institute of Physics, Ke Karlovu 5, Prague 2, CZ-121 16, Czech Republic

[d] School of Physics, Peking University, Yiheyuan Road No.5, Haidian District, Beijing, China

[e] Department of Chemistry, City University of Hong Kong, Kowloon 999077, Hong Kong

donato.valli@kuleuven.be, heng.zhang@physik.uni-regensburg.de, majo.betusiak@gmail.com, gr@chem.ku.dk, arne.meulemans@kuleuven.be, daniel.escudero@kuleuven.be, sudipta.seth@kuleuven.be, zhaoqing@pku.edu.cn, zonglzhu@cityu.edu.hk, bonn@mpip-mainz.mpg.de, eduard.belas@matfyz.cuni.cz, roman.grill@matfyz.cuni.cz, wanghai@mpip-mainz.mpg.de, johan.hofkens@kuleuven.be, elke.debroye@kuleuve.be

**Table S1.** Lattice parameters and cations ionic radius for pristine and doped samples. Data reported from ref. [1–4]. *Radius estimated from calculations (Figure S1).

| Material | Lattice parameter (Å) | Ionic radius (Å) |
|---|---|---|
| $Im^+$ | 11.200 ± 0.004 | 2.58 |
| $G^+$ | 11.170 ± 0.003 | 2.78 |
| $A^+$ | 11.140 ± 0.004 | 1.46 |
| $T^+$ | 11.190 ± 0.003 | ~2.44* |
| D($Cs^+$/$Bi^{3+}$) | 11.25575 ± 0.00023 | 1.81/1.03 |
| $Ce^{3+}$ | 11.25059 ± 0.00023 | 1.03 |
| $Eu^{3+}$ | 11.25773 ± 0.00034 | 0.95 |
| $Gd^{3+}$ | 11.25997 ± 0.00028 | 0.94 |
| $Er^{3+}$ | 11.26115 ± 0.00022 | 0.881 |
| $Cr^{3+}$ | 11.25575 ± 0.00023 | 0.62 |

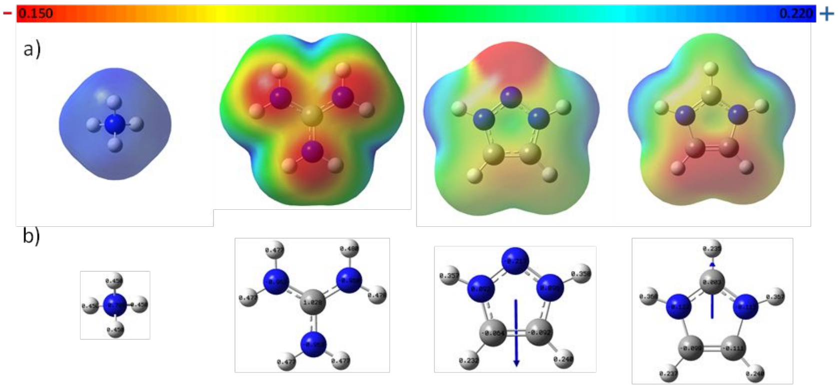


**Figure S1.** (a) Color-coded (from 0.150 to 0.220 a.u., top bar) electrostatic surface potentials (ESPs) for the different ammonium cations (at an electron density isocontour of 0.00040 a.u.), from left to right: ammonium, guanidinium, triazolium, and imidazolium. (b) Corresponding partial atomic charges and dipole moments.

## Optical characterization

The bandgap variations observed among the different substitutions, including the redshifts in $Eu^{3+}$-, $Er^{3+}$- and ammonium cations doped samples and the blue shifts in $Ce^{3+}$- and $Gd^{3+}$-doped ones, were too minor to significantly impact X-ray sensitivity. Additionally, for the aromatic cations, the shift in bandgap follows lattice contraction, with a smaller lattice parameter leading to a lower bandgap.

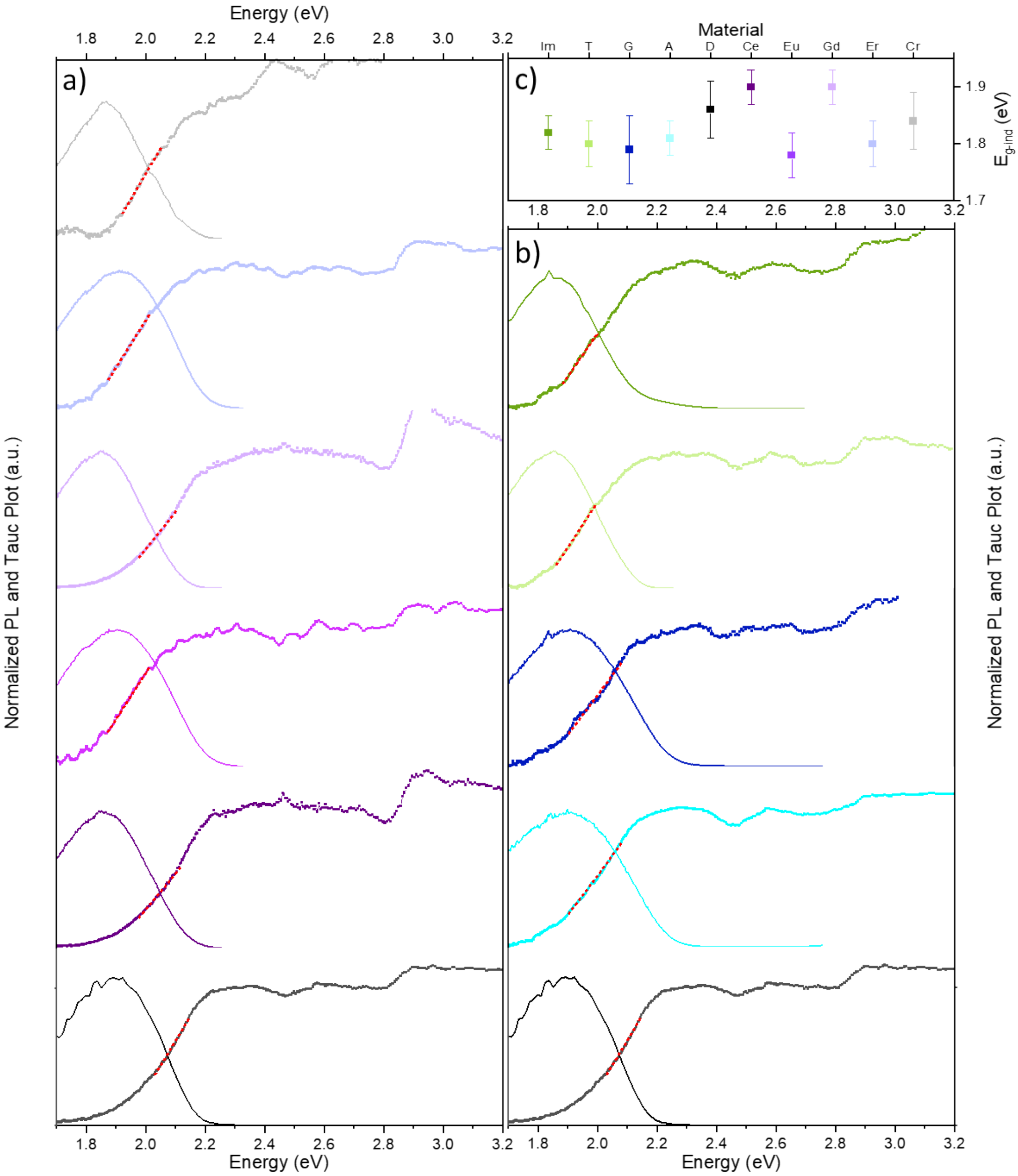


**Figure S2.** Comparative analysis of pristine, **D,** and doped samples. **(a, b)** Tauc plots (squared dots connected with a line as a guide to the eye) and photoluminescence (PL) spectra (solid lines) for the pristine and doped samples, indicating negligible differences in their steady-state optical properties. The resulting indirect bandgap energies, extracted from the linear regression of the band edges (red dotted lines), for both the pristine and doped materials are depicted in **(c)**.

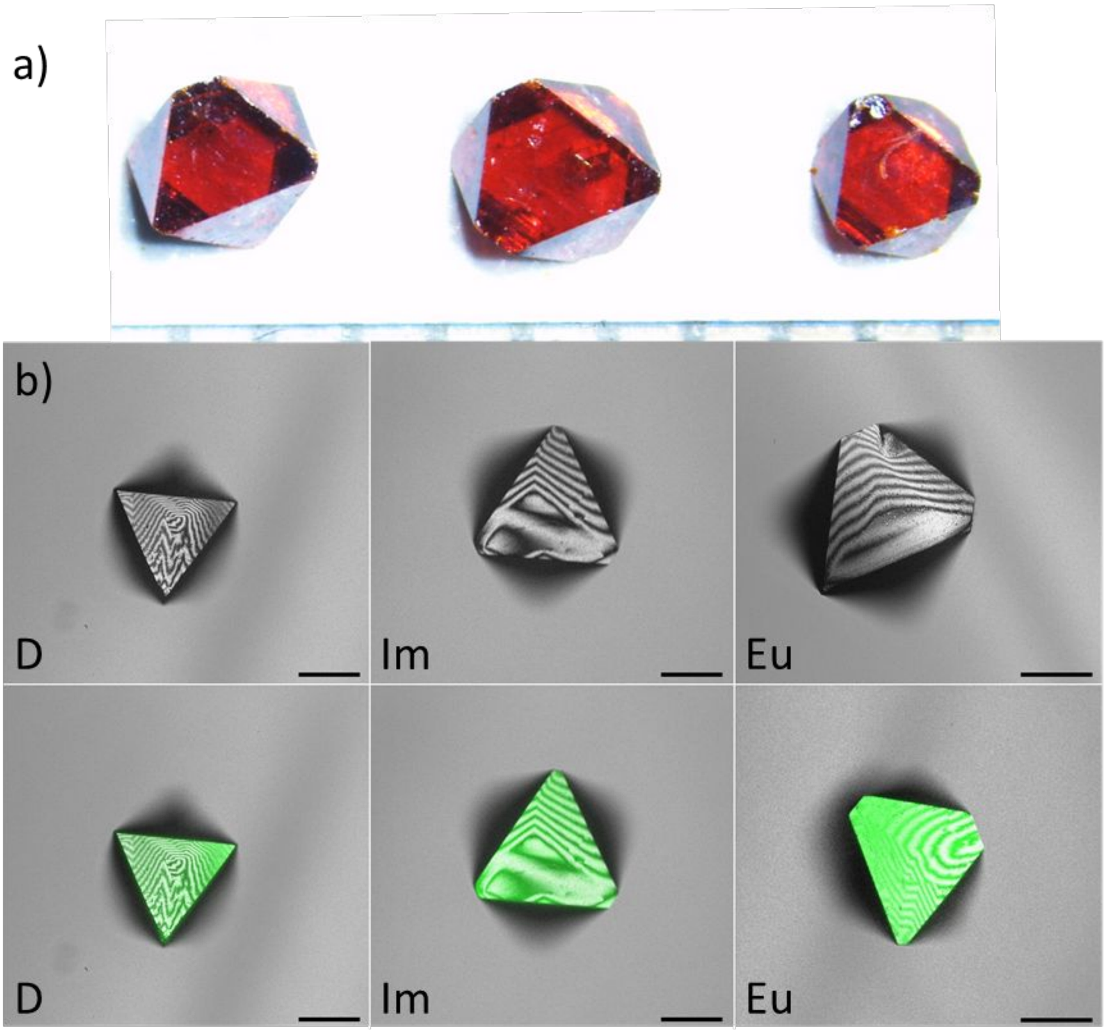


**Figure S3.** (a) Optical image of representative single crystals for the pristine (left), Im (middle) and Eu materials (right). (b) Confocal microscopy images of the microcrystalline counterparts, showing (top) overlap between reflection and transmission images to give a 3D appearance to the materials and (bottom) superimposition of the resulting PL. The black bar corresponds to 100 µm. No drastic change in PL intensity has been noted between the different materials under 405 nm laser excitation at 65 µW laser power.

**Model of photoconductivity evolution**

We have observed that the evolution of photocurrent after X-ray excitation is relatively slow process lasting for about tens of second (Figure S4a). In contrast, less resistive samples show nearly abrupt jump to new value (Figure S4b). We explain this phenomenon by an existence of deep mobile donors with ionization level positioned below or close to Fermi energy in high-resistive samples. Logically, in less resistive sample the Fermi energy localizes closer to the valence band and the level is mostly ionized. Consistently with general belief, we expect that all samples are of p-type conductivity and neglect participation of electron at the electric transport. After the irradiation, the photo-excited holes are trapped on the level increasing this way the density of mobile charge. Since this process takes some time, the gradual rise of $I_{ph}$ is seen. We show in Figure S4c the scheme of levels outlining

principal processes occurring in high resistivity and low resistivity irradiated samples.

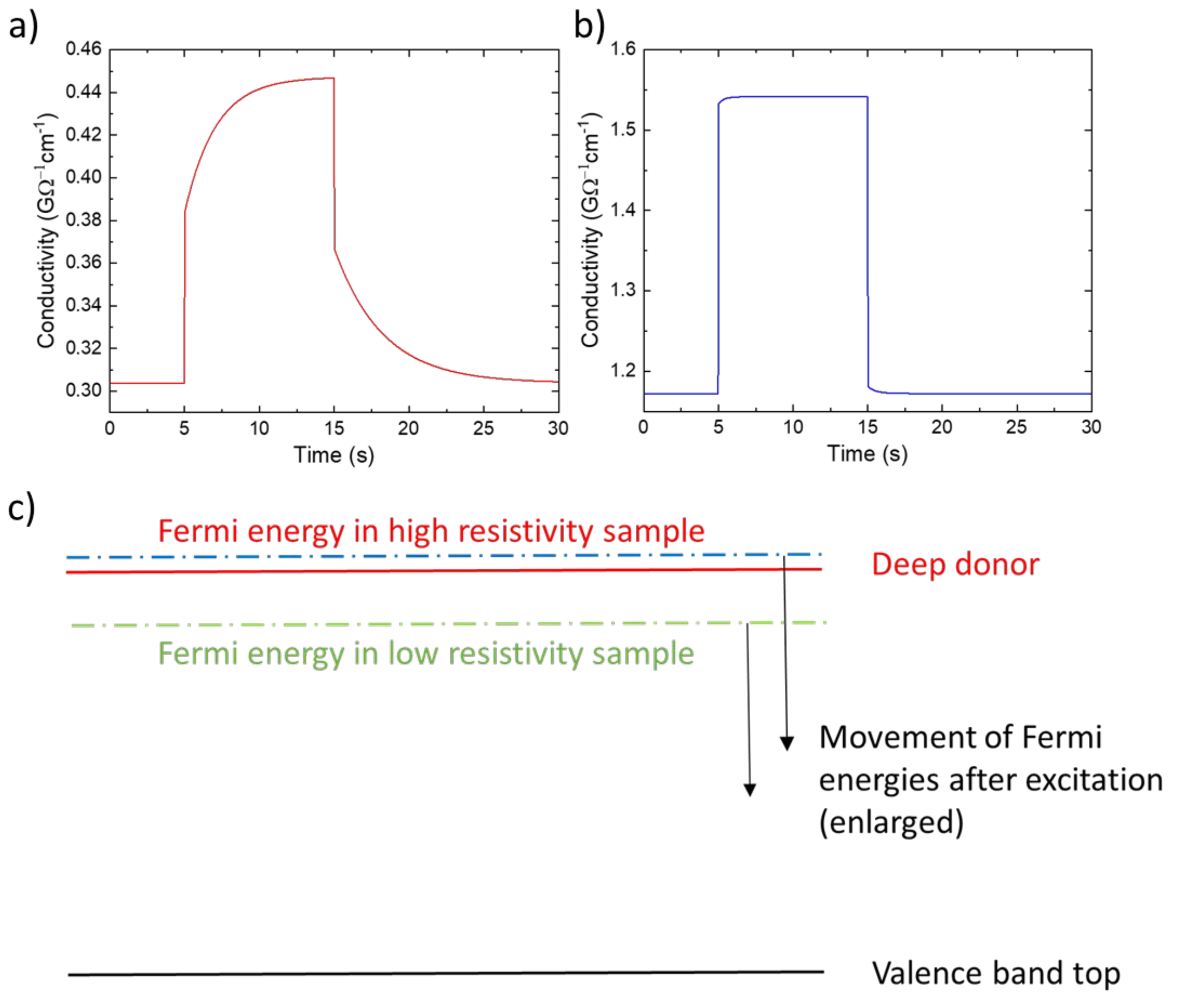


**Figure S4.** Simulation of conductivity relaxation after periodic X-ray excitation in the high resistivity (a) and low resistivity (b) samples. c) Corresponding energy scheme of the Fermi level shifts in high resistive and low resistive samples.

**Table S2.** K-edge energy for the different lanthanides dopants[5] and Bi[6].

| Element | Atomic number | K-edge energy (KeV) |
|---|---|---|
| Ce | 58 | 40.4 |
| Eu | 63 | 48.5 |
| Gd | 64 | 50.2 |
| Er | 68 | 57.5 |
| Bi | 83 | 90.5 |

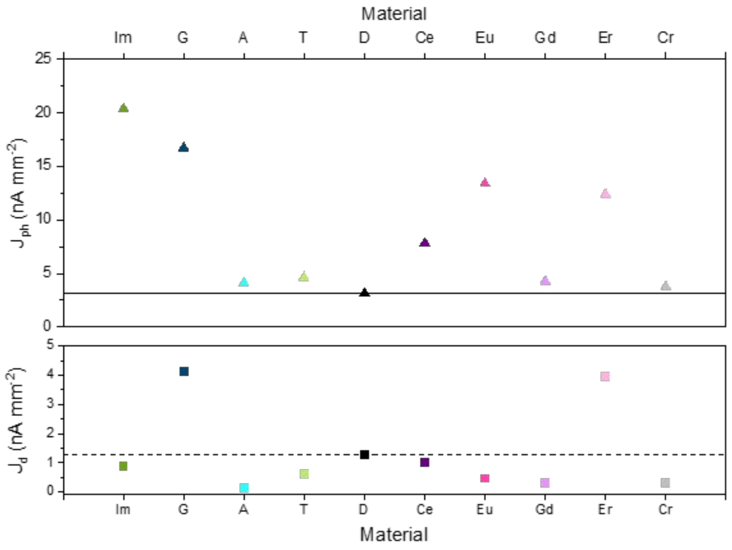


**Figure S5.** Comparative analysis of pristine, **D** (black dots), and doped samples $J_d$ (square) and and $J_{ph}$ (triangle), at 10 V bias showing that all the dopants increase $J_{ph}$ compared to **D** while for the **G** and **Er** there was also an increase in $J_d$ as compared to **D**.

## Reference


(1) Kieslich, G.; Sun, S.; Cheetham, A. K. Solid-State Principles Applied to Organic–Inorganic Perovskites: New Tricks for an Old Dog. *Chem. Sci.* **2014**, *5* (12), 4712–4715. https://doi.org/10.1039/C4SC02211D.

(2) Cotton, F. A.; Wilkinson, G.; Murillo, C. A.; Bochmann, A. *Advanced Inorganic Chemistry, 6th Edition*, 6th Edition.; Wiley-Interscience: New York, 1999.

(3) Ouyang, R. Exploiting Ionic Radii for Rational Design of Halide Perovskites. *Chem. Mater.* **2020**, *32* (1), 595–604. https://doi.org/10.1021/acs.chemmater.9b04472.

(4) Saliba, M.; Matsui, T.; Seo, J.-Y.; Domanski, K.; Correa-Baena, J.-P.; Nazeeruddin, M. K.; Zakeeruddin, S. M.; Tress, W.; Abate, A.; Hagfeldt, A.; Grätzel, M. Cesium-Containing Triple Cation Perovskite Solar Cells: Improved Stability, Reproducibility and High Efficiency. *Energy Environ. Sci.* **2016**, *9* (6), 1989–1997. https://doi.org/10.1039/C5EE03874J.

(5) Tsang, M.-K.; Bai, G.; Hao, J. Stimuli Responsive Upconversion Luminescence Nanomaterials and Films for Various Applications. *Chem. Soc. Rev.* **2015**, *44* (6), 1585–1607. https://doi.org/10.1039/C4CS00171K.

(6) Kim, J.; Bar-Ness, D.; Si-Mohamed, S.; Coulon, P.; Blevis, I.; Douek, P.; Cormode, D. P. Assessment of Candidate Elements for Development of Spectral Photon-Counting CT Specific Contrast Agents. *Sci Rep* **2018**, *8* (1), 12119. https://doi.org/10.1038/s41598-018-30570-y.
